# Dr. Webster on Epidemic Cholera

**Published:** 1832-10-01

**Authors:** 


### An Essay on Epidemic Cholera. By *John Webster*, M.D.

In this cheap and portable little volume is concentrated a great mass of information respecting the present epidemic. Dr. Webster took an active part in the investigation of the disease during its first visitation in this metropolis, and, therefore, his observations are entitled to great respect. He has all along maintained liberal opinions respecting the propagation of the epidemic, and has taken great pains to compare it with former diseases, and to investigate its supposed contagious characters. We can only introduce a short extract as a sample of the work; and that will be best selected from a long and able chapter, entitled "References to English Medical Authors." Dr. Webster, in a preceding chapter, examines the ancient writers, and also those of the Continent, after which he comes to the English authorities. This chapter, as, indeed, the whole work, evinces considerable research and patient investigation.

"Ancient records, of epidemical diseases, prevailing in England, during the early ages, are so meagre in the detail of symptoms, that it is almost impossible to ascertain them with any accuracy; as for instance, it is difficult, if not impracticable, to find out the prominent features, distinguishing the pestilence of 1348, already mentioned, as having prevailed in London, and where 100,000 people died from the disease; it having committed besides, great ravages in Europe and in Asia. In this latter part of the world, so great was the devastation, that priests, physicians, and all classes of society, perished; nay, the very birds of the air also died; and the living were scarcely able to bury the dead. This visitation of so mortal a disease, is considered, both by some English, and foreign authorities, notwithstanding the imperfect information possessed, to have been the cholera. Had the same minute description of the phenomena, characterizing the disease, the collateral circumstances accompanying, and the pathological changes supervening, in those cases, proving fatal, been but handed down to posterity, in the same accurate manner, as future ages will obtain from the present generation, relative to the more modern epidemick; then, little or no difficulty would have existed, fully to establish the similarity of this, or any other ancient pestilential visitation, with that of more recent occurrence. Unfortunately, such facilities do not exist, and, therefore, we can only conjecture, what were the symptoms, from the few imperfect records remaining.

But these doubts do not apply, excepting to the most ancient medical writers of this country; for the more modern, and in a most eminent degree, the accurate Sydenham, cannot be herein comprehended. This celebrated author, contains in his works, a description of cholera morbus, which by most physicians in this country, has been more frequently referred to, than any other, during the cholera controversy. The description, like that of all other diseases, coming under the notice of Sydenham, is clear, distinct, and not to be misunderstood; and as the complaint, of which he treats, was of more frequent occurrence, and more fatal in its consequences, during the period he lived in, than it had been for some time before, or has since appeared, until the present prevailing complaint; it may be considered, as constituting a similar disease, to the one he describes; whilst many readily include them both, under the same classification.

This cholera morbus, we are told by Sydenham, was more epidemic in the year 1669, than he had ever remembered to have known it, at any other period; generally coming on, about the close of Summer, or towards the beginning of Autumn; as constantly, he observes, as swallows in the beginning of Spring, and cuckoos towards Midsummer. There is also an indisposition, caused by a surfeit, according to Sydenham; which happens at any time of the year, resembling with respect to its symptoms, the cholera morbus, yielding to the same treatment as it does, but its species, he observes, is yet different. The true cholera morbus is, however, according to this accurate author, easily known, by the following symptoms. 'Immoderate vomiting, and a discharge of *vitiated* humours by stool, with great difficulty and pain; violent pain and distension of the abdomen, and intestines; heartburn, thirst, quick pulse, heat and anxiety; with frequently, a small and irregular pulse; great nausea, and sometimes colliquative sweats; contraction of the limbs, fainting, coldness of the extremities; and other like symptoms, greatly terrifying the attendants, and which often destroy the patient in twenty-four hours.'

At the close of the Summer of 1676, the same cholera morbus, we are likewise informed by Sydenham's account, 'raged epidemically, and being rendered more severe by the extraordinary heat of the season, was accompanied with more violent and inveterate symptomatic convulsions, than he had ever before observed. For, not only, the abdomen, as is usual in this disease, but all the muscles of the body, and especially, those of the arms and legs, were affected with terrible spasms, so that, the patient would sometimes leap out of bed, and extend his body different ways, in order, if possible, to mitigate their violence. To exemplify these observations, Sydenham describes a case of a person, to whom he was called at this time, along with his intimate friend, Dr. Goodal. This patient, Sydenham states, was reduced to the last extremity, by the symptoms above mentioned, as characterizing the cholera morbus; attended 'with excessive vomiting, cold sweats, and a scarce perceptible pulse.' Cases of the same disease, this author likewise met with, at all seasons of the year.

Feeling anxious to know the degree of mortality, this epidemic cholera morbus exhibited, and which Sydenham mentions, was more extensive and severe, than he had ever before known, a reference was accordingly made, to the bills of mortality for the year 1669; but no death whatever, is reported in these documents, under the name of cholera morbus. Indeed, it should as well now be stated, that this appellation, so commonly used, in the works of Sydenham, and of most other medical authors, was not adopted, in the bills of mortality, for more than a hundred years afterwards; nor is it found in any preceding register. However under the head of 'Gripings in the Guts,' some years previously designated 'Plague in the Guts;' there are entered, in the burials within the bills of mortality, during the year alluded to, of 1669, the very large number of 4,385, as dying of this complaint; undoubtedly the vulgar name, for the more scientific one, of cholera morbus, as used by Sydenham, And this in a population of not much more than 600,000, constitutes a very considerable mortality, for one disease. During the following year, or 1670, it is reported 3,690 likewise died of the same disease. The number of deaths, by this complaint, afterwards gradually fell off, and at the beginning of last century, when the population of London amounted to 674,000, they were of a very small amount; and in 1758, only fifty are reported to have died of this malady, so extensively fatal in the time of Sydenham.

The next authority referred to, on this question, is Morton, considered the ablest physician, and most learned member in the medical profession of his day, and a worthy successor of Sydenham; he flourished at the end of the seventeenth century. In one of this author's works, published in 1693, we find the following general outline, of the symptoms, characterizing the cholera morbus, of that period. In this disease, he observes, 'There are present enormous vomitings, and dejection of corrupted humours, from the bowels, with great difficulty and suffering; vehement pain of the stomach and intestines, cardialgia, thirst; pulse quick, frequent, small, and unequal; there is also heat and anxiety, most insupportable nausea, sweat, contractions of the legs and arms, fainting, or deliquium, the extremities are frigid, with similar symptoms, which kill in twenty-four hours.'

But perhaps, the most valuable information, we can obtain, from the classical works of this experienced physician, as they may well be called, being written in excellent Latin, is the detail of some cases, occurring in his own practice, about the time already stated. Particular instances are much more important, than any general description, as that may be derived from the writings, or observations of others; but here, actual cases speak for themselves; and they constitute facts, the most stubborn, whilst they establish points in the discussion the most important, and which reasoning or sophistry, never can explain away, or overcome.

However, before quoting these instructive cases, it will be useful, and may serve to elucidate the subject, if a short digression be allowed, from the point more immediately under consideration, in order to observe, that Morton, when describing the intermittent fever, of his time, says: 'this disease sometimes assumes the type of cholera morbus.' In another paragraph he remarks, 'When the poison,' meaning of intermittent fever, 'participates at the same time of an emetic and cathartic nature, the patient is affected with frequent vomiting and purging, from whence cholera morbus is formed ! Again, in another page, we find this instructive, and most important observation. 'If from the poison,' that is also as regards intermittent fever, 'attacking at the same moment both the stomach and intestines, and from these parts producing a discharge of colliquative humours ; there is then observed enormous vomitings and dejections, along with horrible spasms ; the same as occur in cholera morbus.'

The first case, thought advantageous, here to quote, is the one marked number four, in Morton's publication relating to proteiform diseases. The patient was a servant to a druggist, named Hall, living at Pye Corner, and it is dated the 4th of May, 1691. 'Three hours after dinner, this person was taken suddenly ill, with most painful spasms of the intestines, and enormous vomiting, of different coloured bilious matters, which continued that night, with hiccup, and spasms of the stomach. The diarrhœa remained, accompanied with horrible spasms of the whole abdomen ; latterly, the tormina of the intestines and stomach were most violent, and the vomited matter consisted of a porreaceous, blackish, thin liquor, accompanied with constant hiccup and nausea.' This patient recovered, notwithstanding the violence of his symptoms by the treatment Morton recommended.

The next is that of a young girl, aged 16, previouslyin good health, of elegant appearance, and very clever, as the Doctor expresses. She was taken ill on the 28th of May, and seen by the author in consultation, on the 30th. She lived in Paternoster Row, was named Thatcher, and is the seventh case reported. When visited, this patient was found to be overcome with incessant vomiting, and she was crying out from the horrible spasms she felt in the thorax and abdomen ; and appeared as if about being suffocated. Notwithstanding the violence of these symptoms, there was scarcely any fever, the urine was clear and thin, but nothing is said of its smell. The temperature of the body was low, the extremities were cold, the pulse weak, quick, and irregular. Next day, the symptoms were even more violent, the young lady was almost suffocated, and delirious, from the severe and excruciating pains in the thorax and abdomen ; she threw herself about in the bed, constantly vociferated, and the vomiting continued. This individual likewise ultimately recovered.

It has been remarked, Morton frequently observed cholera morbus to accompany paroxysms of intermittent fever, and many cases in support of this opinion are stated in his work relating to that disease. The example, which may at present be mentioned, is the eighth case ; namely, that of Mr. Arvis. This individual, after returning from the country to London, was suddenly seized with the most acute cholera morbus, according to the author's account, very early in the morning, he having been called up to visit the patient. Morton found him affected with vehement spasms and evacuations, both upwards and downwards, so that the life of the sufferer was in the greatest danger. The extremities were cold, the pulse weak, quick, and almost extinguished, there was deliquium, whilst the other symptoms were most severe.

Notwithstanding the remarkable cases already quoted, from the writings of Morton, a feeling may perhaps continue to linger, in the minds of readers, indicative of doubt, as to the similarity they may exhibit to those of more modern epidemics. Evidence should of course be always of the elearest and strongest kind, to produce conviction : for where an opinion is hastily formed, or upon slight evidence, it is generally erroneous. To those still sceptical, perhaps, the following extraordinary, and precisely similar history of a case, to more modern occurrences, may assist in directing their judgment, to arrive at a correct conclusion. The subject of it was Mr. Ambler, living at Mile End, a valetudinarian, of spare habit of body, and upwards of fifty years old. It is the first case re-reported, in Morton's 'Exercitationes de Morbis Universalibus Acutis ;' published in 1692.

This individual having one day, during rainy weather, in 1690, sailed a long time on the Thames, was, on his return home, suddenly seized with rigour and horror, to which an unusual feeling of coldness succeeded; during the continuance of these symptoms, the vibrations of the pulse could scarcely be felt. He was affected copiously with vomiting, dejections, and horrible oppression of the stomach, and was attacked as in cholera morbus. The apothecary in attendance attempted to resuscitate the vital spark, almost extinguished, before Morton was called in to consultation. Next morning, he was visited by Morton, and he reports, 'the patient exhibited a complete and well-marked hippocratic countenance, the whole body was cold like a piece of stone (glebæ), and covered with sweat; the *skin*, from the coagulation of the blood, was tinged with a blackish, or (nigredine) swarthy colour; the strength was almost exhausted by his sufferings, and the perpetual evacuation of variously coloured bile by the mouth, and of a fetid whitish (albescentis) liquid like cream per anum. Every symptom indicated approaching death, since the vital principle, or the animal spirits, appeared to be so greatly depressed by the poison! By the aid of medicine, the strength of the pulse was a good deal restored; the blackish colour of the skin began to vanish, and the heat of the extremities to return. (Pulsûs robur, plurimum restitutum sensi, nigredinem cutis evanescentem, calorem in extremis partibus redintegraturam.) Nevertheless, all the symptoms soon re-appeared again, with greater intensity than before; and 'the miserable patient expired, within twenty-fours from the time the disease first commenced.'" 139.

We strongly recommend this interesting little volume to the attention of our readers.

